# The impact of semen testing for Ebola virus RNA on sexual behavior of male Ebola survivors in Liberia

**DOI:** 10.1371/journal.pntd.0008556

**Published:** 2020-09-14

**Authors:** Kathleen Tompkins, Jerry Brown, Sam Tozay, Edwina Reeves, Korto Pewu, Harrietta Johnson, Gerald Williams, Tonia Conneh, Joseph Diggs, Jean DeMarco, Katherine King, Darrius McMillian, Carson Merenbloom, William Fischer, David Alain Wohl

**Affiliations:** 1 The Institute of Global Health and Infectious Diseases at the University of North Carolina at Chapel Hill, Chapel Hill, North Carolina, United States of America; 2 The John F. Kennedy Hospital, Monrovia, Liberia; 3 The University of North Carolina Liberia Project, Paynesville, Liberia; 4 Division of Pulmonary and Critical Care Medicine, The University of North Carolina, Chapel Hill, North Carolina, United States of America; NIAID Integrated Research Facility, UNITED STATES

## Abstract

Sexual transmission of Ebola virus (EBOV) is well established and has been implicated in multiple resurgences during the West African Ebola epidemic. Given the persistence of viral RNA in semen, guidelines from the World Health Organization (WHO) recommend abstinence or condom use for at least 1 year or until two semen PCR tests are negative. To better understand the impact of semen testing on sexual behavior, male EVD survivors were surveyed regarding their sexual behavior before and after semen testing. Of the 171 men who enrolled, 148 reported being sexually active following discharge from an ETU with 59% reporting episodes of condomless sex. At least one semen sample for testing was provided by 149 men and 13 of these men had EBOV RNA detected in their semen. When comparing sexual behaviors before and after semen testing, a positive semen test result had limited impact on behavior. Of those with seminal EBOV RNA detected, 61% reported no change in behavior pre- and post-semen testing with 46% engaging in condomless sex before and after testing and only 1 adopted safer sex behaviors following receipt of a positive result. Similarly, among men with undetectable EBOV in their semen, 66% reported no change in sexual behaviors with semen testing, with 55% forgoing condoms during sex. In only 11% was a negative semen result followed by abandoning condoms. There were no known sexual transmission events of Ebola virus in this cohort despite viral presence in semen during periods of condomless sex. This highlights the need to better understand the infectious potential of viral RNA persistence and determine what constitutes effective counseling for survivors and their partners.

## Author summary

Ebola virus (EBOV) is established as a sexually transmitted infection, however there is limited information on the sexual behaviors of Ebola survivors. We surveyed a cohort of male Ebola survivors regarding sexual activity and condom use and offered semen testing to assess EBOV persistence in semen. We found that a large majority of men who were sexually active prior to contracting Ebola Virus Disease remained sexually active after their recovery with most reporting only intermittent condom use. Semen testing for the presence of Ebola virus had limited impact on sexual behaviors, with most continuing to report engaging in condomless sex whether EBOV was detected or not. This finding has implications for use of semen testing for outbreak control as well as counseling for Ebola survivors and their partners.

## Introduction

The development of acute Ebola Virus Disease (EVD) among female sexual partners of male EVD survivors and the persistence of detectable Ebola virus (EBOV) RNA in semen demonstrate the potential of Ebola as a sexually transmitted infection (STI)[1–4] which has important implications for infection control both during and following outbreaks of this virus.

In the midst of the Ebola epidemic in West Africa, the World Health Organization (WHO) issued guidance for the prevention of sexual transmission of Ebola and recommended semen testing for male survivors beginning three months after recovery and continuing monthly while engaging in safe sexual practices with condom use or abstinence from sex until semen testing is negative on two consecutive occasions[5]. Where semen testing is unavailable, the WHO advises men to abstain from sex for 12 months following recovery. However, subsequently reported data suggest EBOV RNA can persist in the semen of some survivors beyond 12 months[6], with viral RNA detected more than two years following recovery in a small number of EVD survivors[7]. In addition, intermittent detection of EBOV RNA in the semen has been reported, including a positive test following two negative tests, potentially complicating current WHO recommendations[7].

While numerous studies have highlighted the duration, patterns, and significance of viral RNA detection in semen, there have been comparably few studies that have characterized the sexual behavior of survivors[8]. This study examines the sexual behavior of male EVD survivors in Liberia enrolled in a longitudinal cohort in which men over the age of 18 were invited to have semen tested for the presence of EBOV RNA to determine the impact of EBOV semen testing on sexual practices.

## Methods

Men, women and children who were survivors of EVD were enrolled into the Liberian Longitudinal Ebola Survivor Cohort between June 2015 and June 2016. The cohort was created at the Eternal Love Winning Africa (ELWA) Hospital in Monrovia, Liberia, the site of two large Ebola Treatment Units (ETUs) to assess the longer-term physical and emotional consequences of EVD as well as the persistence of EBOV RNA in male and female genital secretions. The study is supported by the National Institute of Allergy and Infectious Diseases (NIAID) at the US National Institutes of Health (NIH). Participants were recruited to the cohort from an Ebola Survivor Clinic established at ELWA Hospital and from the surrounding communities via word of mouth. Cohort entry required written documentation of prior EVD as evidenced by an ETU discharge certificate containing the name of the participant and verified by photo identification. Participants underwent a comprehensive interview every three months to assess physical symptoms, mental health, and stigma. Additionally, individuals 12 years and older were asked questions about sexual behavior and men aged 18 years and older were invited to provide semen specimens for analysis of viral RNA persistence. All study visits and interviews occurred at the UNC-ELWA research site in Monrovia, Liberia.

Questions related to sexual behavior were selected and adapted from a survey developed by FHI360 to assess HIV risk among youth in Liberia [9]. Participants were asked if they were sexually active, and if so, the number of sexual partners and the use of condoms. Men were asked to report on sexual activity since discharge from the ETU and in the previous 30 days including sexual activity with other men (MSM activity). Individuals who reported not being sexually active were asked to select reasons for not engaging in sexual activity from multiple-choice responses, with the option to choose “other” and provide a free-text response. Participants received $US30 for completion of study surveys.

All surveys and pre- and post-test counseling were conducted in private rooms in Liberian English by research assistants who had received training on Good Clinical Practice (GCP) and at least four hours of training on survey administration. Additionally, they all successfully completed Collaborative Institutional Training Initiative (CITI) ethics and compliance certification.

Detailed information on semen collection and analysis has been described previously [7]. Briefly, samples were collected at 3-month intervals and testing for EBOV RNA was performed using the Cepheid Xpert Ebola Assay (Sunnyvale, CA) to detect Zaire EBOV total nucleic acid, targeting the glycoprotein and nucleoprotein genes [10]. Based on a prior validation study, samples were considered positive if either of the target genes was detected. The results of the semen testing were shared with the participant soon after they became available. Participants were counseled on the investigational nature of the test and that a negative result should not be interpreted to mean no virus was present or that the participant was not potentially infectious given the lower limit of detection determined during assay validation. They were further advised to follow the Liberian Ministry of Health recommendations for Ebola survivors on condom use and abstinence, which were adapted from the WHO interim recommendations[5] and included correct and consistent condom use until two negative tests are received. Participants who tested positive were advised to abstain from sexual activity or use condoms with all sexual encounters until they received further testing. Condoms were provided to all participants. Regardless of semen EBOV RNA test result, the study protocol called for a second sample to be collected within 14 days for confirmation.

At baseline, sexual practices since discharge from the ETU and condom use in the prior 30 days were assessed. For men who provided semen for EBOV testing, we assessed sexual behavior in the 30 days prior to semen testing and in the 30 days prior to their subsequent survey, which occurred a median of 90 days later (range 57–146 days).

### Ethics statement

This study was approved by the Institutional Review Boards of the University of North Carolina at Chapel Hill and the University of Liberia—Pacific Institute of Research (UL-PIRE). All participants provided written informed consent, with assent obtained for minors and consent from their parent/guardian. As per above, participants had to be at least 18 years of age to be eligible for semen analysis.

## Results

Of the 326 people enrolled in the Liberia longitudinal Ebola survivor cohort, 180 (55%) were male. Demographic characteristics of the male participants are shown in Table 1. At baseline, 171 (95%) of the males were aged 12 years and older (and thus had data collected on sexual activity), and their mean age was 34 years (Range 13–68 years). One hundred sixty-six of the 171 (97%) had reached sexual debut and, of these, 148 (89%) reported being sexually active since discharge from the ETU. Of the 148 men who reported sexual activity since ETU discharge, more than half (87/148; 59%) reported having sex without a condom during this period. Male participants had an average of 386 days from ETU discharge to study enrollment (IQR 325 days to 442 days) and 75 were within 1 year from ETU discharge. At study entry, the number of reported sexual partners since discharge from the ETU ranged from 1–20, with a mean of 2 partners and a median of 1 partner. No individuals reported MSM activity.

**Table 1:** Demographic characteristics study participants, N = 171.

Age at study entry (years)	Number (%)
12–17	5 (3%)
18–25	42 (25%)
26–39	76 (44%)
40–59	46 (27%)
60+	2 (1%)
Relationship status at study entry	
Not currently in a relationship	24 (14%)
Not married but in a relationship	104 (61%)
Married	33 (19%)
Widowed	8 (5%)
Divorced/separated	2 (1%)

At the time of study entry, 23 of the 171 male participants reported not being sexually active since leaving the ETU with 5 of these reporting never having had sex. Among those who were sexually active prior to EVD the most common reasons for abstinence were “I have had no interest” followed by “I wanted to but did not have a partner”. No participants cited difficulty achieving erection, feeling too tired or sick, partner refusal, or fear of transmitting Ebola to their partner. Five of these 23 men (22%) reported wanting to engage in sexual activity but were told not to by a healthcare provider because they previously had Ebola.

### Results for those with positive semen testing for EBOV

Semen was donated at least once by 149 men and all but 7 participants provided 2 or more samples for analysis. The median time from ETU discharge to first semen donation for all men was 659 days (range 272–822 days). Thirteen (9%) of those who donated semen provided at least one specimen that tested positive for EBOV RNA. At the time of study entry, 12 of the 13 men whose semen had detectable EBOV at any time point during study follow-up reported being sexually active since their discharge from the ETU and 6 (50%) of these sexually active men with detectable EBOV RNA in semen reported episodes of condom-less sex since ETU discharge.

At the time of the first semen donation (median 288 days from study entry), all 13 participants reported being sexually active. Comparisons of reported sexual activity during the periods immediately prior to positive semen testing and immediately following receipt of a positive result are shown in Table 2. For the majority (8/13; 61%), semen testing was not associated with a change in condom use or abstinence. Of the 13 men who tested positive for EBOV in semen, 6 (46%) reported episodes of condomless sex both before and after their positive test result. An additional 2 men (15%) reported consistent condom use both before and after their positive testing. For the remaining 5 men, a change in sexual behavior was observed following seminal EBOV testing but in only one was this change from higher risk to lower risk behaviors (condomless sex to abstinence).

**Table 2:** Sexual behavior of men before and after semen testing for EBOV.

Behavior before and after semen testing	Semen EBOV positive N (%)	Semen EBOV negative N (%)
No behavior change		
Sex without condoms→sex without condoms	6 (46%)	75 (55%)
Consistent condom use→Consistent condom use	2 (15%)	12 (9%)
Abstinence→abstinence	0 (0)	3 (2%)
Behavior change		
Consistent condom use → Any sex without condoms	2 (15%)	15 (11%)
Consistent condom use →abstinence	1 (7%)	3 (2%)
Abstinence→ Consistent condom use	1 (7%)	5 (4%)
Abstinence→Any sex without condoms	0 (0)	5 (4%)
Sex without condoms→abstinence	1 (7%)	4 (3%)
Sex without condoms→ Consistent condom use	0	11 (8%)
Data missing	0	3
Total	**13**	**136**

### Results of those with negative semen testing for EBOV

**Fig 1:**
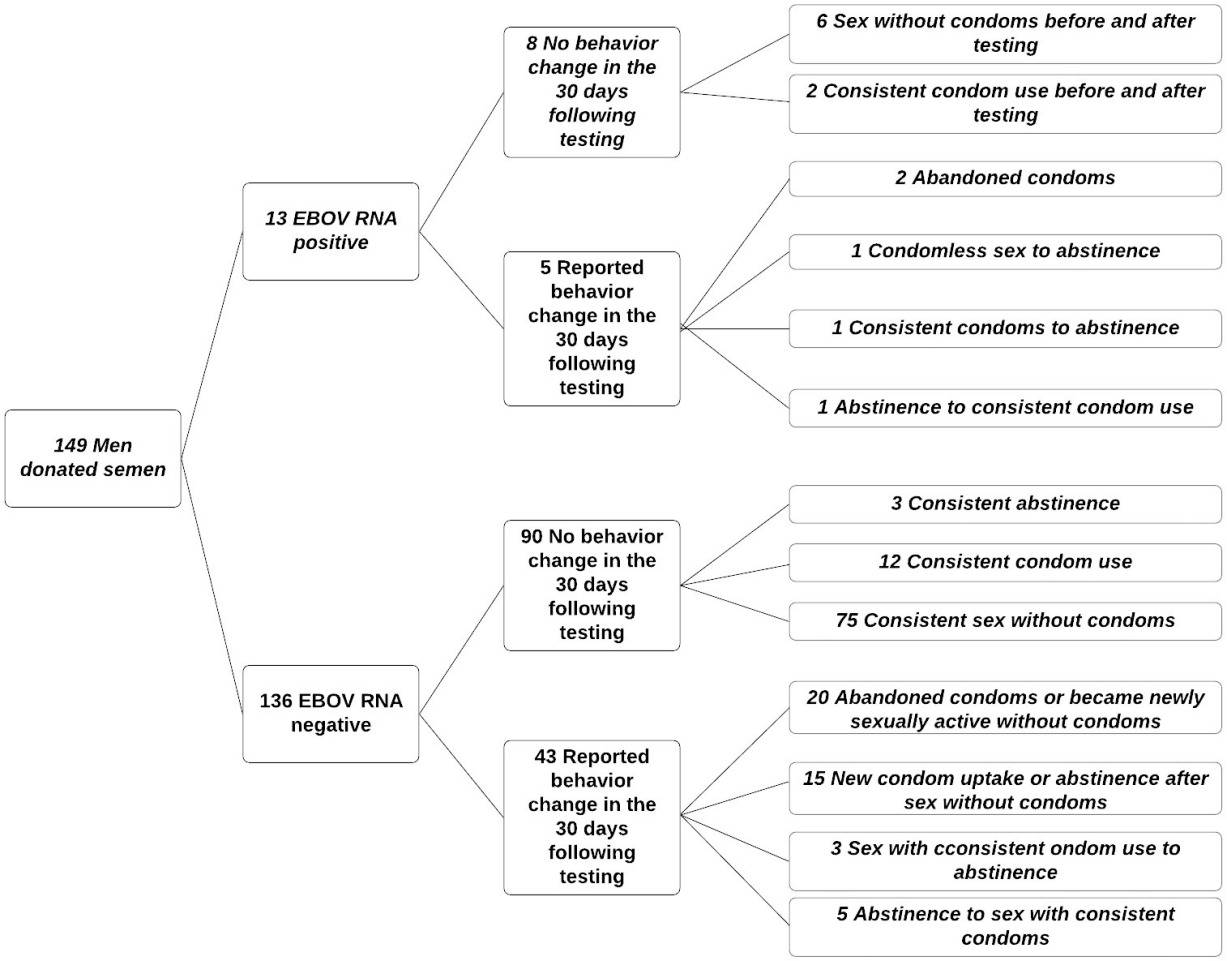
Results of semen testing and subsequent behavior change.

Of the 136 men who provided at least one semen sample and never had EBOV RNA detected, the majority (90/136; 66%) reported no change in sexual behavior, with most of these men (75/136; 55%) reporting episodes of condomless sex both before and after testing negative (Fig 1). Fifteen of 136 men (11%) reported a behavior change from always using condoms in the 30 days prior to semen testing to having at least one episode of sex without a condom in the survey conducted immediately after having negative semen testing. In contrast, 13 of 136 (13%) reported a change from less safe to more safe sexual practices following receipt of a negative semen test result. Responses did not quantify the number of sexual encounters.

## Discussion

In this large cohort of male Ebola survivors in Liberia we found that the majority of those who were sexually active prior to EVD continued to be sexually active after their discharge from the ETU and that more than half reported having sex without a condom. Importantly, in this study semen testing for the presence of EBOV had limited effect on condom use whether EBOV was detected or not detected. In particular, 6 of the 13 men who had EBOV detected in their semen reported condomless sex both before and after testing positive. Two men who were using condoms prior to testing positive stopped using them after receipt of their test result, suggesting a positive result did not encourage condom use. For 5 of the 13 men with detectable EBOV RNA in semen, consistent condoms or abstinence was practiced, however many of these men reported sexual activity without a condom on prior surveys suggesting they did not all consistently adhere to the recommendations. Collectively, this highlights the challenges in dissemination and implementation of guidance on the prevention of sexual transmission of EBOV and emphasizes the need to develop effective strategies to mitigate the risk of sexual transmission of EBOV.

Similarly, most of the men whose semen testing did not reveal EBOV RNA also reported little change in sexual behavior, with most reporting condomless sex both before and after semen EBOV testing. Only 15% either abandoned condoms or became sexually active after being abstinent following the testing, perhaps influenced by the negative results despite the caveats that accompanied the test report detailing the limitations of the assay.

Our finding of a limited impact of semen testing on sexual behavior stands in contrast to a report detailing the results of the Liberian national semen testing program for EVD survivors[11]. That study found rates of reported condom use (defined by condom use at last sexual encounter) increased with semen testing. It is noteworthy that nationwide, Liberia has lower rates of condom use than most other countries in West Africa, with 29.4% of individuals reporting condom use with their last high-risk sexual encounter[12], defined as sex with a non-cohabitating partner. It is possible that the national semen testing program was sought out by those concerned about the possible transmission of EBOV to their partners and that counseling delivered during the visit encouraged condom use. Additionally, the national semen testing program tested men earlier in recovery (median 384 days) than we did in our cohort, which may have contributed to higher rates of condom uptake. Co-enrollment into the national cohort was permitted but not tracked.

In our cohort, a small number of men reported being sexually abstinent at study entry and lack of desire was the most commonly cited reason. We did not probe as to the underlying reason for lack of desire or if this impacted participants’ quality of life, however it is possible that ongoing symptoms or mental health post-EVD may play a role in sexual health. It is notable that on study entry only 5 participants reported not engaging in sexual activity on the advice of healthcare personnel given their history of EVD, despite 75 of 171 men being less than 1 year from ETU discharge. None of the men reported abstaining from sex due to concerns for transmitting EBOV. This finding underscores the importance of developing sexual health strategies and survivor guidance that acknowledges that most men will remain sexually active. Such strategies should be developed with active input from the survivor community and implemented in a manner that does not add additional stigma to this already marginalized group. Traditional approaches to reducing the risk of STIs, may be a useful starting point to prevent EBOV infection of the partners of male survivors, particularly early in convalescence when the risk of transmission appears to be greatest. Studies conducted to enhance condom use to prevent HIV infection have shown success with interventions targeting heterosexual men through group and individual counseling, peer educators, and mass national communication campaigns;[13] however there is evidence to suggest that individuals in long-term or monogamous relationships are less likely to use condoms than those in casual partnerships.[14] The large proportion of individuals in our cohort who reported being in long-term relationships may have contributed to the overall low condom use. Engaging the partners of EVD survivors in couples counseling regarding risk of transmission and condom use may prove beneficial in increasing condom uptake, as has been shown in HIV-serodiscordant couples [15], [16].

The continued sexual activity of male survivors and inconsistent use of condoms we observed has a number of implications. Foremost, the risk of transmission posed by the persistence of EBOV RNA in semen requires greater scrutiny. While viral RNA has been detected in the semen of men two years or more out from acute EVD, in all the documented cases of sexual transmission of the virus, the male source was within 16 months from ETU discharge[6]. In our study, men more than a year past acute EVD who had persistent detection of EBOV RNA in their semen reported condomless sex, yet no female partner is known to have been infected with EBOV—suggesting a possible decline in transmission potential or attenuation of virus hampering transmission. Additional studies to determine the infectiousness of semen containing EBOV RNA are needed.

As discussed, real-time semen testing for EBOV RNA was not found to generally influence behavior in our study but may have an impact when offered as part of routine care and when coupled with other interventions such as counseling and the provision of condoms, as had been shown in other settings[17]. Counseling provided by our team at the time of testing emphasized the research nature of the test and we continued to encourage condom use for all participants. It is possible this was confusing for participants and may have blunted the impact of testing on behavior change. However, semen testing, as described by the WHO, is intended to provide information that allows an individual to calibrate his risk of transmitting EBOV to a partner. As such, abandonment of abstinence and/or condoms can be expected after negative testing, and while in our study only 15% of men with an undetectable EBOV RNA semen result followed such a pattern, it is important to recognize the limitations of EBOV RNA testing of semen including variability in the lower limit of detection across assays and an incomplete understanding of the threshold below which infection is not possible—information that was shared with participants via post-test counseling with opportunities to ask questions to ensure understanding. We and others have also shown that detection of EBOV RNA in semen can be intermittent and can follow repeatedly undetectable tests[7], [18]. In particular, 5 of the 13 men in our cohort who ultimately tested positive had an initial negative test.

While the relatively large sample size, and standardized surveying and semen testing for EBOV RNA are strengths of our study, there were several limitations that should be considered when interpreting our findings. Questionnaires were administered in-person and although conducted by trained staff, there is the possibility of social desirability and/or recall bias. Despite this potential for under-reporting of risk behavior, more than half of the participants reported condomless sex. Additionally, questions on condom use assessed if there were any episodes of condomless sex since ETU discharge and in the past 30 days. This approach may have missed more complex patterns and trends in condom use over time. We also did not determine if participants with more than one sexual partner were using condoms differently with each partner, for example less commonly using condoms with their primary partner but consistently using them with casual partners, as has been found in prior studies related to condom use and other STIs.[19–21] Lastly, a substantial proportion of participants entered the study a year or more after ETU discharge—past the one-year period during which the WHO advises abstinence or condom use. However, of the 148 men who were sexually active on study entry, 73 (49%) were less than a year into their recovery from EVD. Additionally, reports of persistence of seminal EBOV RNA beyond a year were widely known in the survivor community.

While there has been considerable investigation of the dynamics of EBOV in genital compartments, considerably less attention has been paid to the sexual behaviors of EVD survivors. We found that most men in this cohort of EVD survivors remained sexually active with high rates of condomless sex after discharge from ETU and that testing semen for the presence of EBOV had limited impact on condomless sex, including among those with positive EBOV detected. The continued sexual activity of men who survive EVD underscores the importance of determining their actual potential to transmit the virus. Such data are critical to developing evidence-based recommendations and policy to prevent sexual transmission of Ebola and approaches to counseling that will allow survivors and their partners to make informed decisions.

## References

[1] Deen G. F. et al, “Ebola RNA Persistence in Semen of Ebola Virus Disease Survivors—Final Report,” *N Engl J Med*, vol. 377, no. 15, pp. 1428–1437, 10 2017, 10.1056/NEJMoa1511410 26465681 PMC5798881

[2] Mate S. E. et al, “Molecular Evidence of Sexual Transmission of Ebola Virus,” *N Engl J Med*, vol. 373, no. 25, pp. 2448–2454, 12 2015, 10.1056/NEJMoa1509773 26465384 PMC4711355

[3] Fischer W. A. and Wohl D. A., “Confronting Ebola as a Sexually Transmitted Infection,” *Clin Infect Dis*., vol. 62, no. 10, pp. 1272–1276, 5 2016, 10.1093/cid/ciw123 26936667 PMC4845792

[4] Thorson A., Formenty P., Lofthouse C., and Broutet N., “Systematic review of the literature on viral persistence and sexual transmission from recovered Ebola survivors: evidence and recommendations,” *BMJ Open*, vol. 6, no. 1, p. e008859, 1 2016, 10.1136/bmjopen-2015-008859 26743699 PMC4716240

[5] “Interim advice on the sexual transmission of the Ebola virus disease.,” The World Health Organization, Jan. 2016. Accessed: Nov. 13, 2019. [Online]. https://www.who.int/reproductivehealth/topics/rtis/ebola-virus-semen/en/.

[6] Diallo B. et al, “Resurgence of Ebola Virus Disease in Guinea Linked to a Survivor With Virus Persistence in Seminal Fluid for More Than 500 Days,” *Clin Infect Dis*., vol. 63, no. 10, pp. 1353–1356, 11 2016, 10.1093/cid/ciw601 27585800 PMC5091350

[7] Fischer W. A. et al, “Ebola Virus Ribonucleic Acid Detection in Semen More Than Two Years After Resolution of Acute Ebola Virus Infection,” *Open Forum Infectious Diseases*, vol. 4, no. 3, 7 2017, 10.1093/ofid/ofx155 29670927 PMC5897835

[8] Abad N. et al, “Development of risk reduction behavioral counseling for Ebola virus disease survivors enrolled in the Sierra Leone Ebola Virus Persistence Study, 2015–2016,” *PLoS Negl Trop Dis*, vol. 11, no. 9, p. e0005827, 9 2017, 10.1371/journal.pntd.0005827 28892490 PMC5593175

[9] McCarraher D. R. et al, “Informing HIV prevention efforts targeting Liberian youth: a study using the PLACE method in Liberia,” *Reprod Health*, vol. 10, no. 1, p. 54, 12 2013, 10.1186/1742-4755-10-54 24107301 PMC3853775

[10] Loftis A. J. et al, “Validation of the Cepheid GeneXpert for Detecting Ebola Virus in Semen,” *J Infect Dis*., p. jiw562, 12 2016, 10.1093/infdis/jiw562 27932614 PMC5965086

[11] Soka M. J. et al, “Prevention of sexual transmission of Ebola in Liberia through a national semen testing and counselling programme for survivors: an analysis of Ebola virus RNA results and behavioural data,” *The Lancet Global Health*, vol. 4, no. 10, pp. e736–e743, 10 2016, 10.1016/S2214-109X(16)30175-9 27596037

[12] Liberia Institute of Statistics and Geo-Information Services—LISGIS, Ministry of Health and Social Welfare/Liberia, National AIDS Control Program/Liberia, and ICF International, Liberia Demographic and Health Survey 2013. Monrovia, Liberia: LISGIS and ICF International, 2014.

[13] Elwy A. R., Hart G. J., Hawkes S., and Petticrew M., “Effectiveness of Interventions to Prevent Sexually Transmitted Infections and Human Immunodeficiency Virus in Heterosexual Men: A Systematic Review,” *Arch Intern Med*, vol. 162, no. 16, p. 1818, 9 2002, 10.1001/archinte.162.16.1818 12196079

[14] Foss A. M., Hossain M., Vickerman P. T., and Watts C. H., “A systematic review of published evidence on intervention impact on condom use in sub-Saharan Africa and Asia,” *Sexually Transmitted Infections*, vol. 83, no. 7, pp. 510–516, 12 2007, 10.1136/sti.2007.027144 17932124 PMC2598651

[15] Burton J., Darbes L. A., and Operario D., “Couples-Focused Behavioral Interventions for Prevention of HIV: Systematic Review of the State of Evidence,” *AIDS Behav*, vol. 14, no. 1, pp. 1–10, 2 2010, 10.1007/s10461-008-9471-4 18843530 PMC3640442

[16] King R. et al, “Effect of Couples Counselling on Reported HIV Risk Behaviour among HIV Serodiscordant Couples by ART Use, HIV Status and Gender in Rural Uganda,” *PLoS ONE*, vol. 10, no. 9, p. e0136531, 9 2015, 10.1371/journal.pone.0136531 26384103 PMC4575207

[17] Abad N. et al, “Development of risk reduction behavioral counseling for Ebola virus disease survivors enrolled in the Sierra Leone Ebola Virus Persistence Study, 2015–2016,” *PLoS Negl Trop Dis*, vol. 11, no. 9, p. e0005827, 9 2017, 10.1371/journal.pntd.0005827 28892490 PMC5593175

[18] The PREVAIL III Study Group, “A Longitudinal Study of Ebola Sequelae in Liberia,” *N Engl J Med*, vol. 380, no. 10, pp. 924–934, 3 2019, 10.1056/NEJMoa1805435 30855742 PMC6478393

[19] Reynolds H. W., Luseno W. K., and Speizer I. S., “The Measurement of Condom Use in Four Countries in East and Southern Africa,” *AIDS Behav*, vol. 16, no. 4, pp. 1044–1053, 5 2012, 10.1007/s10461-012-0146-9 22307821 PMC3743219

[20] Senn T. E., Scott-Sheldon L. A. J., and Carey M. P., “Relationship-Specific Condom Attitudes Predict Condom Use Among STD Clinic Patients with both Primary and Non-primary Partners,” *AIDS Behav*, vol. 18, no. 8, pp. 1420–1427, 8 2014, 10.1007/s10461-014-0726-y 24567031 PMC4108502

[21] Hock-Long L. et al, “Condom Use with Serious and Casual Heterosexual Partners: Findings from a Community Venue-Based Survey of Young Adults,” *AIDS Behav*, vol. 17, no. 3, pp. 900–913, 3 2013, 10.1007/s10461-012-0177-2 22460225

